# Antral Abscess as a Result of Alveolar Abscess

**Published:** 1891-12

**Authors:** M. O. Cooley


ARTICLE II.

### ANTRAL ABSCESS AS A RESULT OF ALVEOLAR ABSCESS.

(Abstract.)

BY DR. M. O. COOLEY.

I am about to discuss with you the treatment of antral abscess as the result of alveolar dental abscess, which we as dentists are most frequently called upon to deal with. With antral abscess, as with other diseases, different practitioners employ different methods by which they arrive at the same results.

I have endeavored to formulate the teachings of my experience, together with what I have read on this subject that seemed practical and to the point, and I give it to you with the earnest desire that you shall discuss it freely.

Before entering into the details of this subject, it may be well to review a portion of the anatomy and physiology of the antrum and the parts associated with it, for the purpose of refreshing our minds.

The antrum of Highmore is an irregular, triangular-shaped sinus, situated in each of the superior maxillary bones, with its base facing the nose, and its apex the malar process. The floor of the orbit is its roof, and the alveoli of the molar teeth its floor; its only opening is a small foramen, starting from its base or nasal aspect, opening into the middle meatus of the nose, and has as its boundaries portions of the turbinated, ethmoid, and palate bones. The size and shape of the sinus varies to quite an extent in different individuals; in fact, there is often a marked difference in the right and left sides of the same individual. It is said that the average-sized antrum will contain about one and a half fluid ounces. It is larger in the male than in the female, having thicker, stronger walls. In an adult it diminishes in size as age advances or if the teeth are lost.

It is not at all infrequent that there are one or more septa of bone in the floor of the antrum, which, in case of disease, add very much to the difficulty of its treatment.

It is lined with a mucous membrane, which is continuous with that of the nose and the mouth. The antrum in a normal state indirectly communicates with the frontal sinus through intermediate cavities, known as the ethmodial aud sphenoidal cells, and the mucous lining of the antrum is also common with these cavities.

The mucous membrane lining these cavities is made up of ciliated cells a provision of nature by means of which the antrum is enabled to rid itself of secretions which would otherwise accumulate and become offensive.

The relationship which exists between the teeth and the antrum has been found by many of us to be a close one, as in many instances the roots of one or more of the teeth penetrate the floor of the antrum.

There are many lesions which the antrum is heir to, but the one most frequently met with is the result of a broken or abscessed tooth, usually a second bicuspid or first molar. There may not be any great amount of pain in the teeth, but there is a sense of fullness, a pressure in the face which, as the engorgement increases, becomes almost unbearable. A sense of fullness and pain is also very pronounced in the frontal sinus.

With these symptoms in view, the first consideration is to remove the root or tooth, which we will suppose has abscessed, the pus from which has found its way into the antrum. This will usually afford an opening through which the accumulation of pus will find an outlet, whereupon the pain in most cases will subside; but the treatment does not end here, for until nature has made the necessary repairs the secretion of pus will continue, and it is imperative that the opening be large enough to acommodate a tube of gold or silver, which shall be at least one-quarter of an inch in diameter. After the drainage-tube is in place, a clasp-plate should be adjusted to the adjoining teeth, which will serve to keep food from finding its way into the antrum.

In cases where trouble has existed for some time prior to treatment, necrosis is very liable to be present, and should be carefully looked for. If there should be any, it is usually found near the tooth or root which has given rise to the trouble, and in this location is easily removed by use of the dental engine. If necrosis is suspected of having made any progress within the antrum, a simple means of detecting it is to stuff the cavity full of absorbent cotton, allowing it to remain until it has swollen by the moisture it will absorb, until it fills the cavity in every part, when it may be removed. If there be no necrosis, the cotton will not pull apart and catch on to the walls of the antrum, as the mucous membrane will be unimpaired and there will be no exposed and rough surface of the bone to become entangled in the fibres of the cotton, as would be the case should necrosis exist. However, anyone with sufficient experience can with a probe readily detect necrosed bone, but I consider the cotton, in the hands of the inexperienced, the safer and more reliable.

Now the question arises, How shall we deal with the necrosed bone? In some cases it may be necessary to make the opening sufficiently large to admit of its removal by surgical means, but in the majority of cases this is not necessary, for by administering systematically a tonic treatment, such as syrup of hypophosphites or cod-liver oil,and wholesome diet, in conjunction with local treatment, which shall be stimulant and disinfectant, the sequestra will be exfoliated and repair will take place without an operation. Our homœopathic brethren claim gratifying results from high potencies of silicia, calcaria carbon and phosphorus.

The progress made in cases of long standing is often very slow, sometimes requiring several months of careful and persistent treatment, while those which are taken in hand in the early stages are by far less obstinate, and the possibility of effecting a permanent cure is proportionally more flattering.

I have no doubt if we were to examine the sockets of all the superior first molars we extract, that the number whose roots are found to perforate the floor of the antrum would astonish us, and we would marvel that there are not more cases of antral abscesses than there seem to be.

I am lead to believe that in not one case in ten, antral abscess follows the extraction of a tooth whose roots perforate the floor of the antrum. The temperament, general health, and recuperative power of the patient have a very strong bearing on this point.

Nasal catarrh offers serious resistance to the treatment of what might otherwise be simple cases to handle, especially in this damp climate. Owing to the catarrh, the mucous membrane of the nose becomes hypertrophied, closing the natural outlet of the antrum, thereby rendering it impossible to close the artificial opening. Moreover, the inflammation is more difficult to control. It is well understood that an inflammation once begun in the mucous tract has a tendency to spread and involve other organs having the same lining.

For example, a child who is erupting teeth has an inflammation, which is at first circumscribed, of the gum-tissue surrounding the tooth about to erupt. Gradually it becomes diffused, affecting the mucous membrane of the mouth and fauces, and in many instances the whole mucous tract, causing diarrhœa ,convulsions, and sometimes death. The mucous membrane of the antrum bears a similar relation to that of the nose. The mucous membrane of the antrum is low in vitality, as a result of the abscess, and has no power to resist the inflammation from the nose. As a consequence we have a very stubborn lesion to deal with.

In his system of oral surgery Dr. Garretson states that "The antrum once fully and fairly opened in the floor region, a practitioner is not to attempt its closure. As a rule it will not close, and the writer's experience impresses him that closure, as a rule almost without exception, is the worst thing that can happen. Once fairly exposed by a break in its floor, an antrum never again physiologically voids itself through the nose, the reason for this being that the ciliated expression of the antral mucous membrane has lost its office as a result of the disease that caused the opening."

This statement does not apply to cases where teeth have been extracted, whose roots have penetrated the floor of the antrum, where the disease existed prior to the extraction of the tooth, for as a rule these cases will readily close, provided the outlet into the nose is unobstructed. Hence the importance of getting the catarrhal affection under control at the earliest possible moment. Situated as we are in the lake region, with its damp climate, catarrh is more or less prevalent, and anything like a permanent cure is to be obtained only by going to a higher and dryer place, *and never returning*. I may have put this somewhat strongly, but I have just been treating a case of this character, and my efforts were unavailing until such a change was made, when improvement began, and in a short time the trouble was apparently over. When patients present themselves for treatment they are usually more or less run down systemically, as the result of suffering, loss of sleep, absorption of pus, and the consequent loss of appetite. They should be placed under tonic treatment, as before described, and should take plenty of exercise in the open air. Their diet should be that which is most wholesome and nutritious.

In the local treatment of this condition, cleanliness is of the first importance. The chances of recovery are far more encouraging where cleanliness alone is observed, than where the most skilful medication is practiced without regard to cleanliness.

The remedy for cleansing purposes which has proven most efficacious in my hands is Marchand's peroxide of hydrogen, thrown into the cavity by means of a syringe. This should be followed by injections of a solution of sulphate of zinc, one or two grains to an ounce of water. If there should be much pain, one or two grains of chloral may be added to each ounce of the solution. There are other remedies which may be equally good, such as a mild solution of carbolic acid in glycerine and water, listerine, or sanguinol, used with an atomizer. Some practitioners advocate the use of sulphuric acid, creosote, nitrate of silver, and other escharotics. There may be cases in which they are indicated, but experience of others has taught me that they do more harm than good. We can easily appreciate that where we have an unhealthy granulation these remedies come in very happily where judiciously applied, their use to be followed by mild stimulant and tonic treatment.

Antral abscess, like chronic periodontitis, demands slow and persistent treatment, with mild, soothing medicaments.—*Dentâl Cosmos.*

